# A Complete Response to Immunotherapy in a Patient with Locally Advanced Squamous Cell Lung Cancer Harboring a Novel *TMEM178B::BRAF* Fusion: A Case Report

**DOI:** 10.3390/diagnostics16060909

**Published:** 2026-03-19

**Authors:** Juan Carlos Redondo-González, Iñigo San Miguel, Marta Rodríguez-González, Juan Carlos Montero, José María Sayagués, Mar Abad Hernández, Emilio Fonseca Sánchez, Edel Del Barco-Morillo, Alejandro Olivares-Hernández

**Affiliations:** 1 Thoracic Oncology Unit, Department of Medical Oncology, University Hospital of Salamanca, 37007 Salamanca, Spain; jcredondo@saludcastillayleon.es (J.C.R.-G.); efonseca@usal.es (E.F.S.); ebarco@saludcastillayleon.es (E.D.B.-M.); aolivares@saludcastillayleon.es (A.O.-H.); 2 Institute of Biomedical Research of Salamanca (IBSAL), 37007 Salamanca, Spain; jcmon@usal.es; 3 Department of Radiotherapy Oncology, University Hospital of Salamanca, 37007 Salamanca, Spain; isanm@saludcastillayleon.es; 4 Department of Pathology, University Hospital of Salamanca, 37007 Salamanca, Spain; martarodriguez@saludcastillayleon.es (M.R.-G.); marabad@usal.es (M.A.H.); 5 Faculty of Medicine, University Hospital of Salamanca, 37008 Salamanca, Spain; 6 Cancer Network Biomedical Research Center (CIBERONC), 28029 Madrid, Spain

**Keywords:** non-small cell lung cancer, *BRAF* fusion, targeted therapies, case report

## Abstract

**Background:** The development of advanced genomic sequencing techniques now makes it possible to identify novel biomarkers and guide the design of targeted therapeutic strategies. For advanced squamous non-small cell lung cancer (NSCLC), V-Raf murine sarcoma viral oncogene homolog B1 (*BRAF*) fusions have not been evaluated as a therapeutic target. However, agents that block the pathway activated by these fusions have shown efficacy in other solid tumors, such as melanoma, astrocytoma, acinar carcinoma of the pancreas, and papillary thyroid tumors. **Case Report:** Here, we present the case of a patient with locally advanced squamous NSCLC and minimal smoking history who was found to harbor a *TMEM178B::BRAF* fusion. Following curative-intent chemoradiotherapy (CRT) and subsequent maintenance immunotherapy, the patient achieved a complete radiological response at 12 months, accompanied by a marked improvement in both quality of life and overall clinical status. **Conclusions:** The findings in this patient underscore the importance of extending molecular genetic studies to patients with squamous histology who lack toxic habits or known risk factors. Gene alterations such as *BRAF* rearrangements may not only predict the response to immunotherapy-based treatments but also represent a promising avenue for the development of new therapeutic strategies.

## 1. Introduction

Lung cancer currently has the highest incidence of any cancer in both sexes, with 2,480,675 new diagnoses in 2022, accounting for 12.4% of all new cancer diagnoses worldwide. In the female subgroup it already ranks second (908,630 cases, 9.4%) [1]. Global statistics estimate that 15% of lung cancers in men and 53% in women are not attributed to smoking, representing 25% of all lung cancer cases worldwide. Historically this subgroup has been under-represented in clinical trials and research studies, but it constitutes a pathological entity with different molecular development. Few reports have explored their structure and impact on clinical course [2].

In recent years, the expanding use of next-generation sequencing (NGS) as a standard of care has enabled the identification of uncommon but actionable alterations found in fewer than 5% of lung cancer patients. Although biomarker research in NSCLC has advanced considerably over recent years, most discoveries have focused on adenocarcinoma, leaving squamous cell carcinoma with limited molecular targets and making it an increasingly important priority for current research efforts. One of the key genes under investigation is *BRAF* (V-Raf murine sarcoma viral oncogene homolog B1), first described as an oncogenic driver in 2002, and a critical signaling molecule in the MAPK/ERK pathway [3]. *BRAF* encodes a RAF kinase downstream of EGFR and ROS1, whose activation promotes uncontrolled cell growth and tumorigenesis, with well-known roles in tumors such as pilocytic astrocytoma, low-grade neuroepithelial tumors, and acinar cell carcinoma of the pancreas.

In NSCLC, *BRAF* alterations, including point mutations, rearrangements, and other less common variants, are found in approximately 4–4.5% of cases overall. In adenocarcinomas, the prevalence is higher, reported to be between 1% and 5%, with many studies centering around 2–4% [4]. Notably, *BRAF* rearrangements represent a particularly rare subset of these alterations, occurring in only 0.2% of NSCLC cases [4]. By contrast, *BRAF* mutations in squamous cell carcinoma are exceedingly rare; for example, in a cohort of nearly 1000 NSCLC patients (646 adenocarcinomas, 231 squamous), no *BRAF* V600E/K mutations were detected in squamous cases [5]. Another study that analyzed the mutational status of *BRAF* in 2001 NSCLC cases found only one squamous carcinoma with a *BRAF* mutation among all *BRAF*-mutated samples [6].

In this paper, we report a patient with locally advanced squamous cell carcinoma (SCC) harboring a *TMEM178B::BRAF* fusion who was treated with concurrent chemoradiotherapy followed by one year of maintenance immunotherapy, achieving a complete radiological response at the end of the treatment and maintaining this response to date, two years after completing therapy.

## 2. Case Report

We present the case of a 54-year-old female patient with no significant medical history, except for her father who died at the age of 70 due to a gastric adenocarcinoma. She was an ex-smoker, with a pack-year index of 7.5 and did not consume alcohol or other toxic substances.

In June 2023, she presented to the emergency department with pleuritic pain in the left hemithorax accompanied by a persistent cough. There were no signs of constitutional syndrome or other associated symptoms, and her laboratory tests were unremarkable. A chest X-ray revealed a left perihilar lesion with increased density.

**Figure 1:**
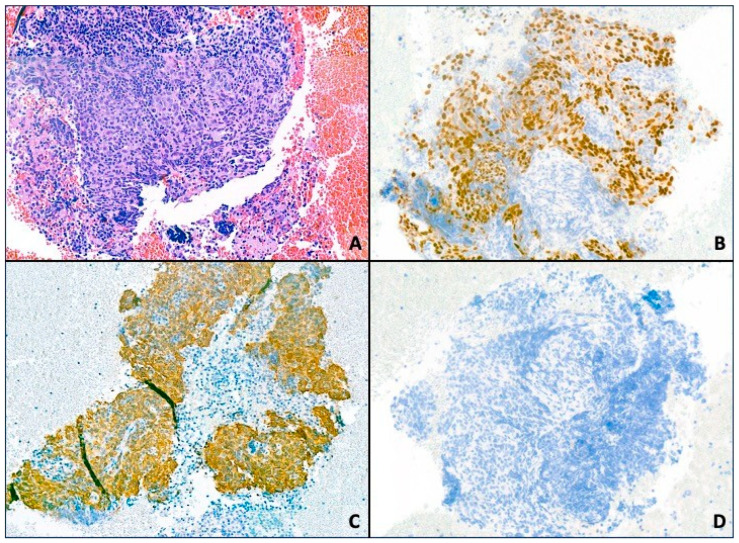
Microscopic images of squamous cell carcinoma diagnosed in the fine-needle aspiration sample. (**A**) Cluster of tumor cells in the hemorrhagic background of the aspiration sample. Hematoxylin–eosin stain. (**B**) Immunohistochemical staining for p40 demonstrating strong nuclear staining in the tumor cells. (**C**) Immunohistochemical staining for CK5 highlighting the tumor cells. (**D**) Immunohistochemical study for TTF-1 negative in the tumor cell population. All images were acquired at 10× magnification.

In the staging study a nodule in the lingula (4.3 × 2.6 cm) was described, compatible with a primary tumor, which was associated with a left perihilar conglomerate measuring 3.5 × 3.2 cm, along with subcarinal and right paratracheal lymphadenopathy measuring 1.7 cm. The tumor was staged as cT2bN3M0 (stage IIIB), confirmed by PET-CT. The sample obtained by bronchoscopy confirmed basaloid-type SCC. The diagnosis was not morphologically straightforward, as the tumor lacked clear features of squamous differentiation such as keratinization, and we therefore relied on immunohistochemistry. The histological immunophenotype was: Cam 5.2+, p40+, TTF1−, and CK7−. CAM 5.2 was mainly used to confirm the epithelial nature of the tumor, although it does not determine the squamous subtype. Tumor cell expression of programmed death ligand 1 (PD-L1) was 40%, determined by the immunohistochemistry assay pharmDX^®^ clone 22C3 in the cell membrane (Figure 1).

By October 2023, a multidisciplinary committee decided on concomitant chemoradiotherapy (CRT) with radical intent and to expand the molecular study using massive genetic sequencing (NGS) due to the low exposure of carcinogenic agents in the patient. Rearrangements at the *BRAF* gene level (*TMEM178B::BRAF*) were detected using an Oncomine Precision Assay (OPA) genomic panel and an Ion Torrent GX5 Chip on a Genexus sequencer (Thermo Fisher^®^, Waltham, MA, USA). The event was classified as a gain-of-function fusion, with 90 supporting reads. No additional concurrent genetic alterations (including SNVs, indels, or copy number variations) were identified.

She began treatment with carboplatin (AUC 5) and paclitaxel (175 mg/m^2^) every 21 days concurrent with radiotherapy, which was completed after two cycles and 33 sessions (total dose of 66 Gy to the primary tumor and lymphatic area). She was unable to receive the third cycle due to hematological toxicities (grade 2 thrombocytopenia, CTCAE 5.0). She achieved a partial response in her December 2023 CT scan and began subsequent maintenance immunotherapy (durvalumab 10 mg/kg every two weeks) 6 weeks after the completion of chemoradiotherapy. However, treatment had to be discontinued due to grade 2 pneumonitis, confirmed by high-resolution CT, which required corticosteroid therapy at 1 mg/kg. After that, the patient was successfully treated for a respiratory tract infection with a regimen of antibiotic therapy based on amoxicillin and clavulanic acid.

**Figure 2:**
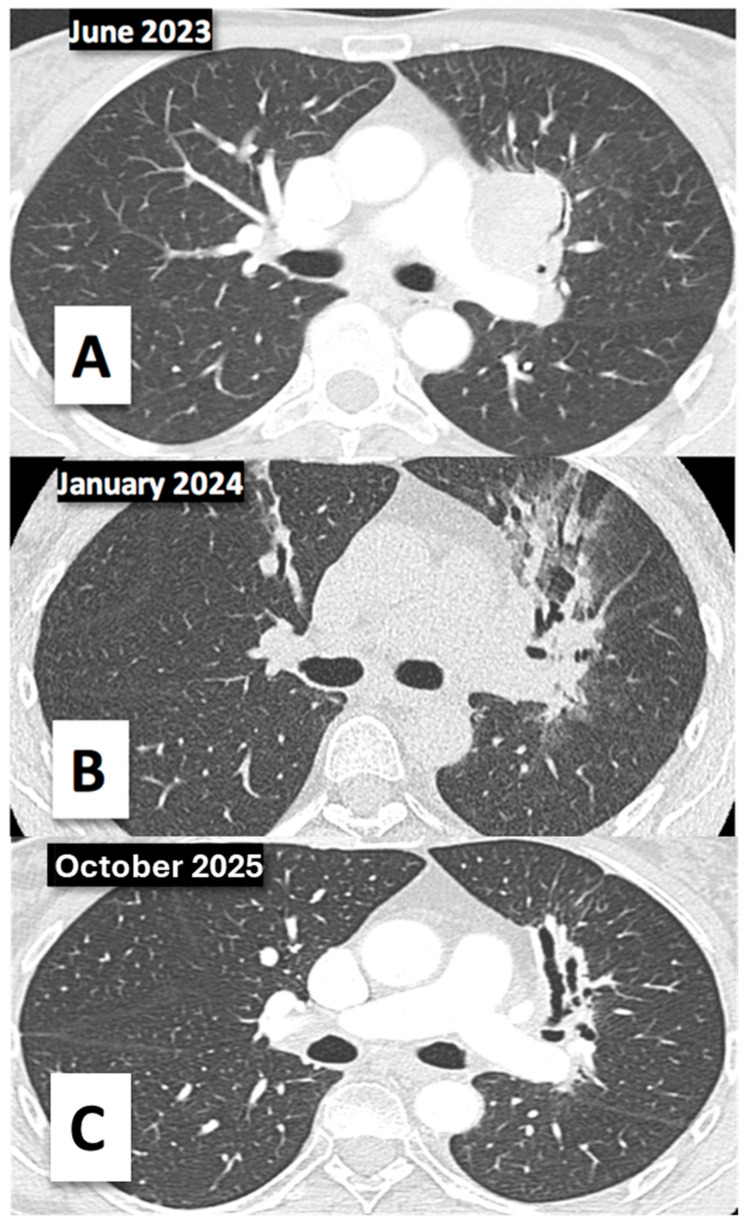
(**A**) Squamous cell lung carcinoma T4N3M0. Stage IIIC. Diagnosis in June 2023. (**B**) High resolution CT scan in January 2024. Peribronchial consolidation and post-radiotherapy pneumonitis and (**C**) complete response maintained in October 2025, with residual signs of post-radiotherapy pneumonitis.

Finally, the patient reached complete radiological response in April 2024, which was maintained at her last re-evaluation in October 2025 (Figure 2). She is currently undergoing quarterly CT scans with an excellent quality of life and is completely asymptomatic, with an ECOG performance status of 0.

## 3. Discussion

In recent decades, a substantial body of knowledge has accumulated regarding biomarkers in NSCLCs. Despite these advances, most clinically relevant biomarkers have been identified in the adenocarcinoma subtype, where more than 1 in 3 patients (33% of cases) benefit from targeted molecular therapies [7].

In contrast, treatment options and prognosis for SCC remain comparatively limited, making this subtype a critical focus of ongoing research. Notably, only 1 in 25 patients (approximately 5%) with SCC exhibit clearly targetable genomic alterations, meaning that just a small subset of this population is able to benefit from targeted therapies [8]. This low prevalence underscores the importance of comprehensive molecular profiling to identify the minority of SCC patients who may still achieve meaningful clinical benefit from precision-based treatments. In this regard, SCC subsets have been characterized by The Cancer Genome Atlas (TCGA) to support the development of new, effective targeted therapies tailored to individual patients [9].

Only 2% of NSCLCs harbor *BRAF* gene alterations, which may represent actionable targets and have the potential to improve clinical outcomes. In our case, NGS was performed individually to identify actionable alterations, given the patient’s young age, squamous cell carcinoma diagnosis, minimal few risk factors and low smoking history (IPA 7.5 pack-years). This analysis revealed a *TMEM178B::BRAF* fusion.

*BRAF* mutations account for approximately 1% of NSCLCs, with the *BRAF* V600E mutation representing around 50% of these cases. This mutation has already demonstrated clinical utility when combined with MEK 1/2 inhibitors for the treatment of various solid tumors [10]. Rearrangements at the level of the *BRAF* gene represent a different pathogenic mechanism. They were first described in 2005 in thyroid cancer, and like point mutations they contribute to aberrant activation of the MAPK signaling pathway [11]. Since then, *BRAF* fusions have been identified across a wide spectrum of solid tumors, suggesting a broader oncogenic role beyond their initial description. In fact, several recent reports have documented *BRAF* rearrangements in diverse malignancies, often associated with variable but sometimes meaningful responses to targeted therapies. Table 1 summarizes the clinical cases published to date, highlighting the heterogeneity of tumor types harboring *BRAF* fusions, the diversity of fusion partners, and the therapeutic strategies implemented. Their potential as a therapeutic target in solid tumors has been recently explored by Ross et al. [12], who reported a discrete representation of all NSCLC cases (around 0.2%). The study highlighted the effectiveness of *BRAF* inhibitors, particularly in spitzoid melanoma, pilocytic astrocytoma, pancreatic acinar and papillary thyroid cancers.

In contrast to NSCLCs with other actionable driver mutations, *BRAF*-mutated NSCLC has been shown to exhibit high susceptibility to immune checkpoint inhibitors. Notably, *BRAF* mutations and their functional classes do not appear to negatively affect the clinical outcomes of advanced NSCLCs and are instead associated with immunotherapy susceptibility [13]. In this context, several published case reports and small series have documented meaningful clinical responses to immune-checkpoint inhibitors in tumors harboring non-V600 *BRAF* mutations. For example, durable responses to anti-PD-1 agents have been described in patients with class II and class III *BRAF* mutations, even in metastatic settings refractory to multiple prior lines of therapy [14,15]. Moreover, retrospective cohorts have suggested that *BRAF*-mutant, particularly non-V600, NSCLCs may exhibit higher tumor-infiltrating lymphocyte densities and immune-active transcriptional signatures compared with other oncogene-driven subsets [16,17]. These observations, although derived from limited sample sizes, support the notion that the functional class of *BRAF* mutation shapes tumor immunobiology and may help guide therapeutic decision-making in advanced NSCLC.

Currently, combinations of dabrafenib–trametinib or encorafenib–binimetinib are indicated for advanced NSCLC with *BRAF* V600E mutation. However, the use of *BRAF* inhibitors such as vemurafenib as monotherapy may paradoxically enhance MAPK pathway signaling in tumors with *BRAF* fusions, making their benefits uncertain. This may be explained by the fact that *BRAF* fusions typically function as Class II *BRAF* alterations (RAS-independent dimers), which accounts for their intrinsic resistance to first-generation BRAF monomer inhibitors. Although case reports have explored targeted approaches, the study by Wang et al. [18] described a novel *VPS41::BRAF* fusion identified in a patient with lung adenocarcinoma using NGS but did not report targeted treatment outcomes.

Recent studies suggest that the activation of the MAPK pathway could regulate PD-L1 expression in tumor cells [19]. This regulation occurs primarily at the transcriptional level, where downstream components of the MAPK cascade, particularly ERK, enhance PD-L1 gene transcription. As a result, tumor cells upregulate PD-L1 on their surface, inhibiting T-cell activity and enabling immune evasion, which is a key mechanism of cancer progression and therapeutic resistance [20].

**Table 1:** Summary of reported cases of *BRAF* fusion in solid tumors, with adult patients receiving systemic therapy.

Case	Date of Publication	Diagnosis	Age (yr)/Gender	BRAF Fusion	Targeted Therapy	Response
Castellano-Damaso et al. [21]	23 May 2024	Low-grade glioma	18/male	*KIAA1549-BRAF*	Trametinib	PR
Zhou et al. [22]	28 March 2024	Tenosynovial giant cell tumor	27/male	*SLMAP-BRAF*	Surgery + Chemotherapy	CR
Yasui et al. [23]	23 February 2024	Lung adenocarcinoma	75/female	*SLC44A1-BRAF*	Trametinib	PR
Hirosi et al. [24]	22 September 2023	Melanoma	71/female	*BRAF-ZKSCAN5*	Dabrafenib/trametinib	PR
Heinrich et al. [25]	26 June 2023	Pancreatic cancer adenocarcinoma	~	*TRIM24-BRAF*	Chemotherapy + ICI	CR
Toshiro et al. [26]	6 November 2022	Melanoma	73/female	*BICD1-BRAF*	ICI	PR
Kong et al. [27]	30 January 2023	Lung adenocarcinoma	53/female	*BTN2A1-BRAF*	iEGFR	PR
Clarck et al. [28]	21 May 2022	Melanoma	19/female	*MYO5A-BRAF*	ICI	PR
Yang et al. [29]	19 August 2022	Lung adenocarcinoma	60/male	*SDN1-BRAF*	Trametinib	PR
Yun-Tse et al. [30]	18 March 2022	Lung adenocarcinoma	67/male	*BRAF-KIAA1549* *MET* amplification	Dabrafenib/trametinib/cacpmatinib	SD
Domen et al. [31]	17 January 2022	Myoepithelial carcinoma	57/female	*AGK-BRAF*	Cobimetinib	PR
Cheng-You et al. [32]	5 November 2021	Lung adenocarcinoma	66/male	*LIMD1-BRAF*	Trametinib	PR
Kervarrec et al. [33]	1 October 2021	Primary melanoma of the lung	55/female	*FNBP1-BRAF*	ICI	SD
Chew et al. [34]	15 April 2021	Melanoma	40/female	*SKAP2-BRAF*	Trametinib	PR
Hasegawa et al. [35]	18 June 2021	Rectal cancer	40/female	*EXOC4-BRAF*	Chemotherapy + iEGFR	PR
Shao-Jie et al. [36]	24 May 2020	Malignant soft tissue tumor	52/female	*CDC42SE2-BRAF* *MET* amplification	Crizotinib	SD
Isaacson et al. [37]	25 February 2019	Urothelial carcinoma	69/male	*NRF1-BRAF*	Trametinib	PR
You-cai et al. [38]	4 March 2019	Lung adenocarcinoma	60/male	*TRIM24-BRAF*	Vemurafenib	PR
Huat et al. [39]	12 February 2018	Cholangiocarcinoma	58/male	*YWHAZ-BRAF*	Chemotherapy	PR

BRAF: V-Raf murine sarcoma viral oncogene homolog B1; ICI: immune-checkpoint inhibitor; iEGFR: epidermal growth factor receptor inhibitor; PR: partial response; SD: stable disease; CR: complete response.

Importantly, *BRAF* fusions, like other activating alterations in *BRAF*, can drive constitutive MAPK pathway activation. Unlike the canonical *BRAF* V600E mutation, *BRAF* fusion proteins signal as RAS-independent dimers that strongly and continuously activate MEK–ERK. This sustained MAPK activation can further amplify PD-L1 transcription, potentially creating a more immunosuppressive tumor microenvironment. Therefore, tumors harboring *BRAF* fusions may exhibit elevated PD-L1 expression and distinct immune-evasive properties, with implications for both targeted therapy and response to immune checkpoint inhibitors. In this sense, understanding the link between MAPK activation and PD-L1 expression can guide the development of more effective combination therapies. Targeting the MAPK pathway (e.g., MEK inhibitors) may decrease PD-L1 expression in tumor cells, thereby enhancing their susceptibility to immune-mediated attack. However, trials focusing on the response to treatments in NSCLC with *BRAF* fusions are still scarce and their results are still limited.

Another possible explanation for the patient’s response to immunotherapy could be a relatively high tumor mutational burden (TMB), which is often associated with increased neo-antigen presentation and improved immune recognition. In addition, favorable characteristics of the tumor microenvironment, such as the presence of infiltrating cytotoxic T cells or a pro-inflammatory cytokine profile, may have contributed to enhancing the therapeutic effect.

## 4. Conclusions

The case presented represents the first SCC harboring a *TMEM178B::BRAF* fusion described in the literature achieving a complete radiological response after immunotherapy treatment. This case highlights the potential value of expanding genetic analysis in these patients. Looking ahead, standardizing the use of comprehensive genomic sequencing will be essential to identify individuals who may benefit from targeted therapies. This approach will help broaden the existing evidence base, particularly in patients with low exposure to carcinogens, and should be considered regardless of tumor stage or histological subtype.

## Data Availability

The raw data supporting the conclusions of this article will be made available by the authors on request.

## Abbreviations

The following abbreviations are used in this manuscript:

NSCLC	Non-Small Cell Lung Cancer
SCC	Squamous Cell Carcinoma
BRAF	V-Raf murine sarcoma viral oncogene homolog B1
MAPK	Mitogen-Activated Protein Kinase
ERK	Extracellular Signal-Regulated Kinase
EGFR	Epidermal Growth Factor Receptor
ROS1	ROS Proto-Oncogene 1
NGS	Next-Generation Sequencing
CRT	Chemoradiotherapy
PD-1	Programmed Cell Death Protein 1
PD-L1	Programmed Death-Ligand 1
CT	Computed Tomography
PET-CT	Positron Emission Tomography–Computed Tomography
OPA	Oncomine Precision Assay
CTCAE	Common Terminology Criteria for Adverse Events
ECOG	Eastern Cooperative Oncology Group
TCGA	The Cancer Genome Atlas
MEK	Mitogen-Activated Protein Kinase Kinase
Gy	Gray
AUC	Area Under the Curve
